# A recursive vesicle-based model protocell with a primitive model cell cycle

**DOI:** 10.1038/ncomms9352

**Published:** 2015-09-29

**Authors:** Kensuke Kurihara, Yusaku Okura, Muneyuki Matsuo, Taro Toyota, Kentaro Suzuki, Tadashi Sugawara

**Affiliations:** 1 Department of Basic Science, Graduate School of Arts and Sciences, The University of Tokyo, Komaba, Meguro-ku, Tokyo 153-8902, Japan; 2 Research Center for Complex Systems Biology, The University of Tokyo, Komaba, Meguro-ku, Tokyo 153-8902, Japan; 3 Department of Chemistry, Faculty of Science, Kanagawa University, Tsuchiya, Hiratsuka, Kanagawa 259-1293, Japan; 4 Toyota Physical and Chemical Research Institute, Nagakute, Aichi 480-1192, Japan

## Abstract

Self-organized lipid structures (protocells) have been proposed as an intermediate between nonliving material and cellular life. Synthetic production of model protocells can demonstrate the potential processes by which living cells first arose. While we have previously described a giant vesicle (GV)-based model protocell in which amplification of DNA was linked to self-reproduction, the ability of a protocell to recursively self-proliferate for multiple generations has not been demonstrated. Here we show that newborn daughter GVs can be restored to the status of their parental GVs by pH-induced vesicular fusion of daughter GVs with conveyer GVs filled with depleted substrates. We describe a primitive model cell cycle comprising four discrete phases (ingestion, replication, maturity and division), each of which is selectively activated by a specific external stimulus. The production of recursive self-proliferating model protocells represents a step towards eventual production of model protocells that are able to mimic evolution.

## Abstract (web summary)

The synthetic production of model protocells, which represent potential intermediates between nonliving material and living cells, may help to explain the origin of cellular life. Here, Kurihara *et al*. develop a giant vesicle-based model protocell that is able to self-proliferate recursively in response to external stimuli.

## Graphical abstract

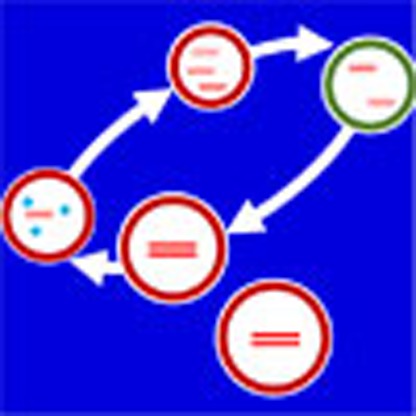

Examination of carbonaceous meteorites^1^, proposed common models and strategies to search for life in the universe^2^,^3^ and synthetic reactions carried out under conditions presumably mimicking prebiotic earth^4^ have prompted continued study of the origin of life on earth. The intrinsic properties of living systems, however, do not come from individual materials, such as nucleotides, peptides and lipids, but from the emergence of collaborative dynamics^5^, such as linked self-proliferation as a model of cell division with chromosomal replication, recursive proliferation with primitive model cell cycles, correlated proliferation between phenotypes and genotypes, and evolution^6^. The aim of our investigation was to explore the universal concept of life by embodying a model protocell that demonstrates how collaborative dynamics emerged from nonliving matter under certain circumstances. To achieve this goal, we selected well-defined suitable lipids and macromolecules, including newly designed ones, and constructed a giant vesicle (GV)-based model protocell that links self-replication of information molecules (RNA/DNA) with the self-reproduction of a compartment (GV)^7^. The membrane of our GV-based model protocell comprises two kinds of phospholipids 1-palmitoyl-2-oleoyl-*sn*-glycero-3-phosphocholine (POPC) and 1-palmitoyl-2-oleoyl-*sn*-glycero-3-phospho-*rac*-(1-glycerol) (POPG), and a synthesized cationic membrane lipid (V) containing an amphiphilic catalyst (C) that catalyses the hydrolysis of the membrane lipid precursor (V*) to yield V and an electrolyte (E) (Fig. 1a). The association between GV self-reproduction and DNA self-replication was mediated by supramolecular machinery comprising a DNA complex with a cationic catalyst (C) buried in the vesicular membrane. This complex served as a pseudo-enzyme that produced vesicular membrane lipids from lipid precursors. The rapid production of membrane lipids at the active site induced a budding-type deformation and eventual GV division in a nearly equivolume manner (Fig. 1b).

An open question in protocell research is how do we establish a protocell that can self-proliferate over multiple generations in a given environment? The term ‘protocell' refers here to a hypothetical precursor of the first cells^8^. They are cell-like compartments, not yet alive, but with many characteristic features of living cells^9^.

To acquire recursive ability, the newborn GV, that is, the daughter protocell, has to restore the status of the original GV through the continual replenishment of depleted substrates for metabolic reactions. This can be problematic for charged or macromolecular substrates that must penetrate the semi-permeable cell membrane. This problem is usually solved by the vesicular fusion technique, which has been explored extensively^10^,^11^,^12^,^13^,^14^,^15^.

Here we show that substrates, such as deoxyribonucleoside triphosphates (dNTPs) and enzymes (DNA polymerase) can be replenished at a specific time during a primitive model cell cycle using a pH-induced vesicular delivery system^16^,^17^, in which the substrate-depleted daughter GV adheres and fuses with a substrate-filled GV. The substrate-filled GV can be regarded as a ‘conveyer' GV, in reference to the fact that vesicular trafficking^18^ is a major transport system for proteins in living cells. We found that four discrete phases (ingestion, replication, maturity and division) emerged spontaneously during our pursuit of a constructive approach towards a recursive model protocell. Our model protocell completed this primitive model cell cycle, in which individual processes in each phase collaborated with the next, specifically responding to external stimuli from the environment.

## Results

### Replenishment of target GVs with dNTPs from conveyer GV

**Figure 1:**
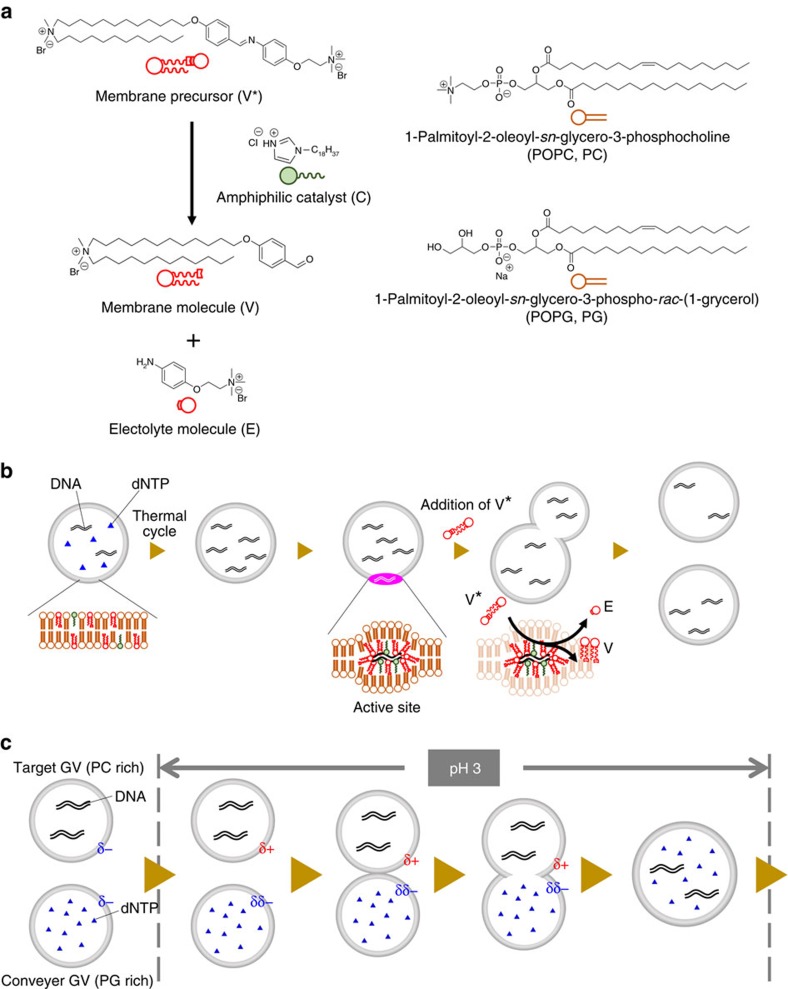
Concept of a self-proliferative GV-based model protocell. (**a**) Membrane lipids consisting of vesicular membrane of self-reproductive GV. Cationic membrane lipid V, amphiphilic catalyst C and phospholipids (POPC and POPG) (right). The membrane lipid V and electrolyte molecule E are generated through the hydrolysis of the membrane lipid precursor V*. (**b**) The production of cationic membrane lipid V from its precursor V*. The cationic membrane V is produced together with the electrolyte E at an active site comprised of amplified DNA and amphiphilic catalyst C in the giant vesicular membrane. The proposed structure of the active site, comprised of amplified DNA, cationic membrane lipid V and amphiphilic catalyst C, for production of membrane lipids is shown in the bottom. (**c**) pH lowering induced adhesion and fusion between the target GV and the conveyer GV. The surface charge of the target GV changes to cationic due to the protonation of the POPC as well as the increase of the cationic membrane lipid V from its precursor, and the target GV adheres to the conveyer GV with a negative surface charge at pH=3. These two types of GVs fuse, and the transport of dNTP from the conveyer GV to the target GV proceeds.

To replenish the depleted substrates of daughter GVs, we constructed a ‘vesicular delivery system' in which a ‘target' GV containing all of the reagents needed for DNA replication, except for dNTPs, adhered and fused with a ‘conveyer' GV filled with dNTPs when triggered by a pH change of the vesicular dispersion (Fig. 1c; Supplementary Fig. 1a; Supplementary Methods). Thus, the daughter model protocell could potentially acquire a sustainable recursive ability to proliferate when external stimuli, such as sequential thermal cycles, and the addition of V* were applied to it.

We simulated the target GVs as newborn daughter GVs, which depleted the substrates (dNTPs). To compare with the membrane composition of the newborn GVs after consumption of membrane precursor, the membrane composition of the target GV was adjusted to POPC:POPG:membrane molecule V:catalyst C:cholesterol=30:7.5:55:5:2.5 mol% (the molecular structures are shown in Fig. 1a). Hence, the surface charge of the target GV was positive. On the other hand, the surface charge of the conveyer GVs (POPC:POPG:catalyst C:cholesterol=25:60:10:5 mol%) that contained dNTPs was negative because anionic POPG was abundant in the membrane. When these two kinds of GVs were mixed and the pH of the vesicular dispersion was lowered, the GVs adhered (Supplementary Fig. 2) and fused during incubation with gentle stirring at pH=3 overnight. Although vesicular fusion between GVs with opposite surface charges has been documented^15^,^19^, this vesicular fusion was triggered by pH lowering because the charge of the POPC of the target GV became positive under the acidic condition (pH=3) produced by the partial protonation of the zwitterionic POPC (p*K*_a_=ca. 1)^20^, whereas that of the conveyer GV remained negative even though almost half of the POPG molecules were protonated (p*K*_a_=ca. 3)^20^. In the current case, the charge of the target GV was positive due to the incorporation of cationic lipid V (Supplementary Fig. 1a), the adhesion might occur even before the acidification of the vesicular dispersion, but the fusion occurred only after the acidification, suggesting that the protonation of POPC is crucial for the membrane reorganization^19^.

After neutralization of the dispersion containing the fused GVs, we subjected the vesicular dispersion to thermal cycles. DNA amplification was clearly detected by assessing the fluorescence emission from a SYBR Green I–double-stranded DNA (dsDNA) complex in the fused GV (Supplementary Fig. 1), suggesting that no deactivation of polymerase occurred due to the proton impermeability of vesicular membranes of the target GV (Supplementary Fig. 3). This observation provided strong evidence for the ingestion of dNTPs by the target GV from the conveyer GV. When the lipid precursor V* was added (Fig. 1b), the fused GVs containing the amplified DNA exhibited a budding-like deformation and subsequently divided (Supplementary Fig. 4). These observations indicate that our GV-based model protocell acquired recursive ability in its self-proliferative cycle. Statistical analysis of flow cytometry data showed that these sequential dynamics, such as adhesion and fusion between the target and the conveyer GVs and division after amplification of DNA using ingested dNTPs, occurred as ubiquitous events (Supplementary Fig. 5; Supplementary Methods).

### Recursive GV-proliferation over three generations

**Figure 2:**
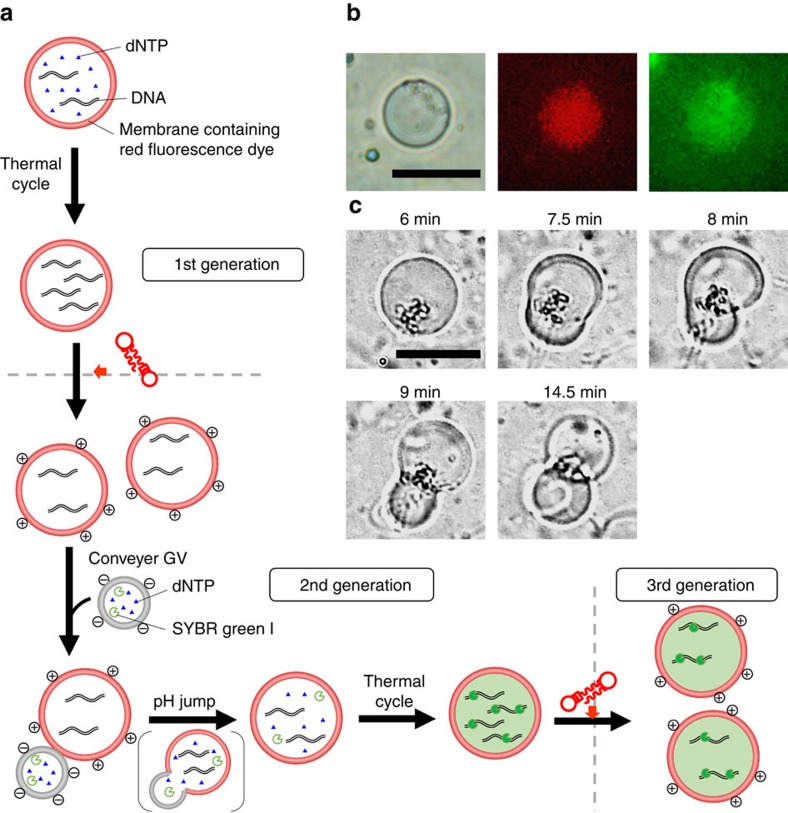
Repeated self-proliferation cycle to produce GV-based model protocell of the 3rd generation. (**a**) Self-proliferation of GV-based model protocell from 1st generation to 3rd generation. DNA amplification in mother GV was followed by the first division to give rise to daughter GVs. Ingestion of dNTP in conveyer GV by daughter GVs and DNA amplification in daughter GV led the second division to give granddaughter GVs (bottom). (**b**) Differential interface contrast microscope image of DNA-amplified daughter GV (left). Fluorescence microscope images of the red fluorescence emitted from the vesicular membrane (center) and the green fluorescence from inside the daughter GV (right). Scale bar, 10 μm. (**c**) Division of the daughter GV to afford granddaughter GVs by the addition of precursor V* of the membrane lipid. Scale bar, 20 μm.

The recursive ability of our model protocell was confirmed by the production of a granddaughter GV in the presence of the vesicular transport system (Fig. 2). The vesicular membrane of the original GV was the same as that used in the previous experiment^7^ and was stained with a rhodamine-tagged lipid (Rhod-DOPE, 0.1 mol%) to distinguish the offspring of the original GV from accidentally formed GVs, whereas the conveyer GVs with the negative surface charge contained dNTPs and SYBR Green I (Fig. 2a). These two types of GVs with opposite charges adhered and fused upon co-incubation, as occurred in the previous experiment (Methods).

After neutralization of the GV dispersion, the GVs were subjected to thermal cycles according to the protocol (Methods). Fluorescence microscopy images of the PCR-subjected GVs showed intense green fluorescence from the dsDNA and SYBR Green I complex inside the GV and red fluorescence from the membranes stained with Rhod-DOPE (Fig. 2b). These data unequivocally demonstrate the amplification of DNA in the fused GV. We already confirmed that most of the mother GVs containing amplified DNA divided into daughter GVs when V* was added, as previously reported^7^ (Methods). Hence, GVs that emitted green fluorescence from the dsDNA and SYBR Green I complex and red fluorescence from the membrane stained with Rhod-DOPE could be accurately designated as daughter GVs bearing amplified DNA (Fig. 2c).

### Significance of the four phases in the self-proliferative GVs

**Figure 3:**
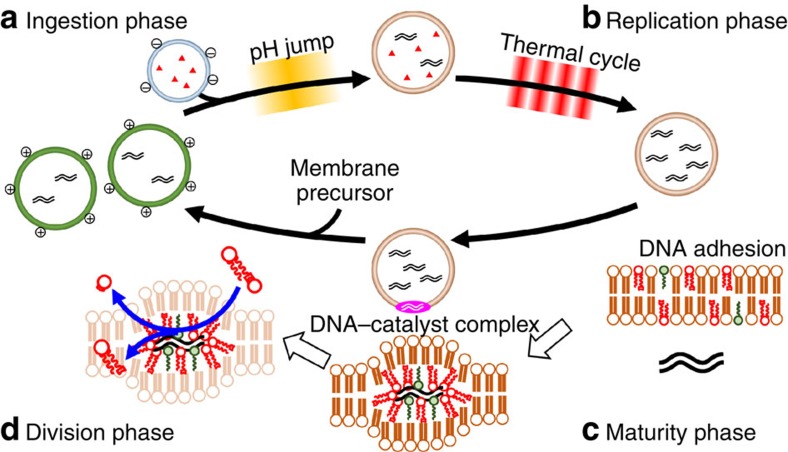
Primitive model cell cycle of self-proliferative model protocell with four discrete phases. (**a**) In the ingestion phase, the GV of the next generation ingests substrates through vesicular fusion with conveyer GV containing dNTP, triggered by a pH jump. (**b**) In the replication phase, the replication of DNA in the next-generation GV proceeds using ingested dNTP. (**c**) In the maturity phase, the catalytic ability of the vesicular membrane matures in a sense that a complex between amplified DNA, amphiphilic catalyst C and cationic lipids V intrudes into the vesicular membrane, forming an active site for converting membrane precursor V* to lipid membrane V. (**d**) In the division phase, the self-proliferative GV grows and exhibits a budding deformation and an equivolume division when the precursor V* of the membrane lipid is added to the exterior of GVs.

This self-proliferative cycle can be divided into four discrete phases: ingestion, replication, maturity and division (Fig. 3), which could be considered as a primitive model cell cycle. These phases are described and discussed in detail below.

In the ingestion phase, the protocell ingests the depleted substrates (dNTPs) via the vesicular delivery system, in which conveyer GVs transport the required substrates to the original (target) GV. This delivery system is dependent on selective adhesion and fusion between GVs with different surface charges (Fig. 3a). We confirmed that this vesicular delivery system could also replenish depleted DNA polymerase (Supplementary Fig. 6; Supplementary Methods), and other reagents that would need to be ingested after several rounds of division. Of note, the membrane composition of GVs varies during the cycle due to the incorporation of cationic membrane lipids derived from their precursors. However, it is possible to restore the membrane composition of the daughter GV, as in the case for the dNTP substrates, by fusing the daughter GV with conveyer GV containing the complimentary lipids. Therefore, it would be a trivial matter for vesicles to oscillate their constitutions through cycles.

In the replication phase, DNA is replicated only in the GVs that have ingested dNTPs through the vesicular fusion that occurred during the ingestion phase (Fig. 3b). DNA replication is driven by periodic changes of temperature (–(94–68 °C)_20_–)^7^,^21^ (Supplementary Fig. 7; Supplementary Methods). Efficient DNA replication depends on the status of the inner cavity of the GVs. Specifically, if the inner cavity of the GV is large and sufficient reagents are encapsulated, the efficiency of DNA amplification is optimal, and DNA is amplified by even a smaller number of thermal cycles^7^,^21^. The amplified DNA in the GV shifts the equilibrium between the adhered DNA on the inner membrane surface and the dissolved DNA in the inner water phase to the adhered side, which prepares the active sites (see next paragraph) for membrane lipid formation within the vesicular membrane. The replication phase ceases when the dNTP is depleted.

The maturity phase is characterized by maturation of the catalytic activity of the vesicular membrane and the DNA–catalyst complex^22^,^23^. This phase is not triggered by an external stimulus as in the ingestion or replication phases; it is activated by an internal stimulus corresponding to an increase in the concentration of the amplified DNA in the inner water pool of the GV. The division frequency of GVs that contain amplified DNA is much greater than that of GVs, which do not contain amplified DNA^7^. This finding strongly suggests that amplified DNA accelerates GV growth and division by the DNA–catalyst complex, which consists of amplified DNA, cationic lipid V and amphiphilic catalyst C. The DNA–catalyst complex intrudes into the vesicular membrane, creating an ‘active site' for the production of membrane lipid V from its precursor V* (Fig. 3c). During the maturity phase, sufficient membrane lipid V is locally provided around active sites. Immediately after the local concentration of V becomes high, the next phase (the division phase) starts. Of note, only GVs in which the active site is constructed can undergo growth and division.

The division phase is the phase of proliferation of the GV containing the amplified DNA after the addition of membrane precursor. The DNA–catalyst complex not only serves as the pseudo-enzyme to produce membrane lipid V from its precursor V*, but also acts as a scaffold for GV division, because a series of divisions occurs around the same position in the vesicular membrane. The divided daughter GVs contain partitioned DNA (Fig. 3d). Only one type of DNA is present in our GV-based model protocell, but the role of DNA in division may be crucial, because the DNA–catalyst complex serves as a pseudo-enzyme that directly participates in division dynamics.

## Discussion

As stated above, our recursive GV-based model protocell demonstrated a primitive model cell cycle comprising four discrete phases. However, the mechanism of division of our model protocell in the division phase seems too simple for a living cell. For example, even *Escherichia coli* express a special protein (FtsZ) that they arrange along the circumference of the cell for compression^24^. It has recently been suggested that even modern bacteria retain the ability to switch to a simple division mode in which a lipid-synthesizing protein is activated to produce excess membrane lipids, leading the elongated cell to divide^25^,^26^. This phenomenon suggests that primitive cell division could potentially proceed without complicated division machinery derived from proteins^27^.

To turn the primitive model cell cycle around, external stimuli, such as thermal cycle in the replication phase or the addition of conveyer GVs with sufficient substrates and pH jump in the ingestion phase, are necessary, which seems to be too artificial treatments for a sustainable model protocell. However, the amplification of DNA could be driven by naturally occurring thermal cycles, such as a convection flow near the hydrothermal vent on an ocean bed^28^. The vesicular transport system could also operate even in a prebiotic environment, because the vesicles could experience the pH gradation around an acidic hydrothermal vent. In this study, we simply simulated such external stimuli by thermal cycles or the addition of an acidic solution^29^,^30^ in the laboratory. While ‘active' GVs, which contained all of the necessary reagents for DNA amplification, were formed by the swelling of thin films of phospholipids within a rich soup, thousands of ‘inactive' GVs that lacked at least one crucial ingredient (for example, template DNA, DNA polymerase) were also generated. Hence, divided GV that lacked dNTPs could fuse with inactive GVs that contained dNTPs, corresponding to conveyer GVs.

To develop advanced model protocells, we must explore the possible correlation between the DNA in the GV (genotype) and the properties of that GV (phenotype). The current GV-based model protocell has the advantage of correlating genotype with phenotype because of the close ‘biological' distance^31^ between these two types, which is brought by the fact that a complex of DNA with the cationic catalyst serves as a pseudo-enzyme. If the genotype of the DNA (for example, the length of the DNA or the ratio of multiple DNAs) in a GV influences the phenotype of a GV-based model protocell (for example, division modes and frequencies), the correlation between these two types may be plausible because the length of DNA in the DNA–catalyst complex may influence the production of membrane lipids, which controls the efficiency of the budding-type deformation and the subsequent division. As a rare event in a round of divisions, a ‘mutant model protocell' containing a specific length or ratio of DNA could emerge during fusion not with the conveyer GVs but with a ‘different species' of model protocell containing different DNA. This cell might exhibit a high frequency of self-proliferation and could become the predominant species in a given environment. Then, this event could be regarded as evolution for the GV-based model protocell. Hence, the construction of a recursive model protocell may represent a first step towards an advanced model protocell, which in some respects could mimic evolution.

## Methods

### Materials

Regents were purchased from the following sources: POPC, POPG sodium salt and 1,2-dioleoyl-*sn*-glycero-3-phosphoethanolamine-*N*-lissamine rhodamine B sulfonyl, ammonium salt (Rhod-DOPE) were purchased from Avanti Polar Lipids, Inc. (AL, USA). The membrane lipid molecule V, amphiphilic catalyst C and membrane lipid precursor V* were synthesized as previously reported^7^. The restriction enzyme EcoRI, pBR322 vector and Wizard SV Gel and PCR Clean-up system for purification of the PCR products were purchased from Promega Corporation (WI, USA). DNA primer 1 (20 mer, 1,363–1,344, 5′-GACAGCATCGCCAGTCACTA-3′) and DNA primer 2 (20 mer, 200–219, 5′-GAGAACTGTGAATGCGCAAA-3′) were purchased from Sigma-Aldrich Japan (Tokyo, Japan). 10,000 × SYBR Green I nucleic acid gel stain and 10,000 × SYBR Gold nucleic acid gel stain were purchased from Life Technologies (CA, USA). 10,000 × SYBR Green I and SYBR Gold were diluted 10,000 times by adding Tris(hydroxymethyl)aminomethane–EDTA buffer (TE buffer) and used as 1 × SYBR Green I and 1 × SYBR Gold. KOD-Plus-(DNA polymerase) and dNTPs were bought from Toyobo Co, Ltd (Osaka, Japan). Size marker DNA and DNase I were purchased from Takara Bio Inc. (Shiga, Japan). TE-saturated phenol–chloroform–isoaminoalcohol (25/24/1, *v*/*v*/*v*) was purchased from Nacalai Tesque, Inc. (Kyoto, Japan). Coprecipitation agent Ethachinmate and sodium acetate (3 M, pH 5.2) for ethanol precipitation were purchased from Nippon Gene Co., Ltd (Tokyo, Japan). Pre-cast 12.5% polyacrylamide gel (e-PAGEL minigel, AE-6000 E-T12.5 L) was purchased from ATTO Corporation (Tokyo, Japan). Other reagents used in this study were obtained from conventional chemical and biochemical suppliers. Preparation of template DNA was prepared as follows: to obtain template DNA (1,164 bp, 200–1,363) for PCR amplification, 1 μg of pBR322 plasmid vector DNA (4,361 bp) was incubated for 16 h at 37 °C in 20 μl of standard restriction digest buffer containing five units of EcoRI. After incubation, the digested pBR322 DNA was amplified by PCR using primers 1 and 2. The amplified 1,164-bp DNA was purified using a clean-up system. The concentration of purified DNA was estimated by measuring absorption at 260 nm.

### Amplification of DNA in GVs

The original GV or the fused GV dispersion mixed with DNase I solution was incubated for 30 min. Next, the GVs containing PCR reagents were placed in a thermal cycler (iCycler, Bio-Rad Laboratories Japan Inc., Tokyo, Japan) for DNA amplification. The thermal conditions were as follows: 94 °C for 2 min, (–(94–68 °C)_20_–). After the thermal cycles were performed, the dispersion was slowly cooled to room temperature.

### Microscopic observation of GVs

The GV dispersion was placed between two glass coverslips with a spacer (17 × 28 mm^2^, 0.3-mm-thick, *In situ* PCR Frame-Seal Incubation Chamber, Bio-Rad Inc., MA, USA). Differential interference contrast and fluorescence microscope images of the GVs were obtained with an optical microscope (IX 70, Olympus, Tokyo, Japan) equipped with a × 20 objective lens and an optical filter set (U-MWIG2; *λ*_ex_: 520–550 nm, *λ*_em_:>580 nm, U-MWIB2; *λ*_ex_: 460–490 nm, *λ*_em_: 510–550 nm).

### Flow cytometric analysis

SH800 (Sony, Tokyo, JPN) was equipped with lasers emitting at 488 and 561 nm for flow cytometry. The intensities of forward light scattering at 488±17 nm and rhodamine fluorescence at 660±30 nm from each dispersion were monitored. IsoFlow (Beckman Coulter, BA, USA) was used for the sheath flow. The GVs were counted at 100,000 per measurement. The flow cytometric data were analysed using SH800 software (SONY).

### Polyacrylamide gel electrophoresis

By means of the previous procedure^21^, PCR products in the GV were retrieved by adding 150 μl of TE-saturated phenol–chloroform–isoamyl alcohol (25/24/1, *v*/*v*/*v*) to the GV dispersion after the PCR and DNase I treatment (150 μl), and the lipids and enzymes were removed from the buffered solution containing the PCR product. The PCR product in the resulting aqueous solution was then purified using the Wizard SV Gel and PCR Clean-Up System according to the manufacturer's protocol.

### Preparation of anionic conveyer GVs

Stock chloroform solutions of the individual lipids (POPC, POPG, amphiphilic catalyst C and cholesterol) were mixed in a test tube to a final molar ratio composition of POPC:POPG:amphiphilic catalyst C:cholesterol=25:60:10:5. The lipid film was prepared as described above. The resulting lipid powder was rehydrated in 1 ml of buffered solution (deionized water (610 μl), PCR buffer (100 μl), MgSO_4_*aq*. (40 μl), polyethyleneglycol (PEG20,000)*aq*. (80 μl), dNTP mixture solution (80 μl), primer 1 (30 μl), primer 2 (30 μl), 1 × SYBR Green I dye (10 μl) and KOD-Plus-(20 μl)).

### GV fusion

After the addition of the conveyer GV dispersion (2 mM, 1 ml) to the newborn GV dispersion (2 mM, 1 ml), the mixed GV dispersion was agitated slowly. Hydrochloric acid (100 mM) was dropped into the dispersion until the pH reached 3, as determined using a pH meter (Handheld Water Quality Meter D-51, Horiba, Tokyo, Japan). After the addition of the hydrochloric acid, the dispersion was stirred and incubated at 23 °C for 1 day. Then, NaOH*aq*. (100 mM) was added to the dispersion until the pH was restored to 8.

### Addition of membrane precursor

The solution of membrane precursor was prepared according to the protocol in previous research^7^. A dispersion of the membrane precursor V* (2 mM) was prepared by dissolving powdered V* in a PCR buffer and sonicating for at least 5 min. The resulting dispersion of V* was mixed with an equimolar dispersion of GVs.

### Model experiment of replenishment of dNTP to target GV

The target GV (POPC:POPG:V:catalyst C:cholesterol=30:7.5:55:5:2.5) and the conveyer GV (POPC:POPG:catalyst C:cholesterol=25:60:10:5) were prepared via the freeze-dry method. The inner solution of the target GV contains deionized water (670 μl), PCR buffer (10 × KOD-Plus-buffer, 100 μl), MgSO_4_*aq.* (25 mM, 40 μl), PEG20,000*aq.* (12.5 wt%, 80 μl), primer 1 (10 μM, 20 mer, 30 μl), primer 2 (10 μM, 20 mer, 30 μl), 1,164-bp DNA template (10 nM, 10 μl), SYBR Green I (1/10,000, 20 μl) and DNA polymerase (0.6 μM, 1.0 units per microlitre KOD-Plus-, 20 μl). The inner solution of the conveyer GV contains deionized water (700 μl), PCR buffer (10 × KOD-Plus-buffer, 100 μl), MgSO_4_*aq.* (25 mM, 40 μl), PEG20,000*aq.* (12.5 wt%, 80 μl) and TE buffer solutions of the dNTP mixture (2 mM per each nucleotide, 80 μl). After the addition of the dispersion of conveyer GV to that of target GV at pH=3, the mixed dispersion was stirred and incubated at room temperature for 1 day. The dispersion was neutralized by adding 0.1 M NaOH*aq.*

### DNA amplification in the GV fused with conveyer GV

To confirm that the amplification of DNA occurred by consuming the conveyed dNTPs, conveyer GVs were fused with the self-reproduced target GV stained by Rhod-DOPE. The membrane composition of the conveyer GV was the same as the conveyer GV as mentioned above. The contents of the inner water phase of the conveyer GV were deionized water (680 μl), PCR buffer (100 μl), MgSO_4_*aq.* (25 mM, 40 μl), PEG20,000*aq.* (80 μl), dNTP mixture (80 μl) and SYBR Green I (20 μl). The amplification of DNA after the fusion was performed by the thermal cycles in the same manner as described above.

### Preparation of 1st generation GVs

Stock chloroform solutions of the individual lipids (POPC, POPG, membrane molecule V, amphiphilic catalyst C and cholesterol; the concentration of each stock solutions is 10 mM) were mixed in a test tube and adjusted to a final molar ratio composition of POPC:POPG:V:C:cholesterol=60:15:10:10:5 (total volume is 100 μl). The mixture was dried to a thin lipid film under a stream of nitrogen. Then the lipid film was stored under reduced pressure for more than 6 h to remove traces of solvent. The dry lipid film was hydrated by the addition of deionized water (1 ml), and then vortexed for 5 s, resulting in the formation of a GV dispersion (total lipid concentration=2 mM). The GV dispersion was incubated for 3 h at 23 °C. It was then rapidly frozen in liquid nitrogen and stored under reduced pressure over a period of 12 h. The resulting lipid powder was rehydrated in 1 ml of a PCR solution (deionized water (610 μl), PCR buffer (10 × KOD-Plus-buffer, 100 μl), MgSO_4_*aq*. (25 mM, 40 μl), PEG20,000*aq*. (12.5 wt%, 80 μl), TE buffer solutions of dNTP mixtures (2 mM per nucleotide, 80 μl), primer 1 (10 μM, 20 mer, 30 μl), primer 2 (10 μM, 20 mer, 30 μl), 1,164-bp DNA template (10 nM, 10 μl), DNA polymerase (KOD-Plus-, 1.0 units per microlitre, Mg^2+^ free, 0.6 μM, 20 μl)). To stabilize the GV, the dispersion was incubated for at least 1 h at 23 °C. After this incubation, a buffer solution containing DNase I (5,000 units per microlitre, 20 μl) was added to the dispersion to digest the DNA template and primers from the exterior of the GV. The outer buffer solution contained deionized water (780 μl), PCR buffer (10 × KOD-Plus-buffer, 200 μl), CaCl_2_*aq*. (100 mM, 10 μl), PEG20,000*aq*. (12.5 wt%, 80 μl), DNase I (5,000 U μl^−1^, 20 μl) and a fluorescent probe for the amplified dsDNA (1 × SYBR Green I, 20 μl). The solution was incubated for 30 min at 23 °C.

### Transportability of conveyer GV

The target GV dispersion contained the following solution: deionized water (630 μl), PCR buffer (10 × KOD-Plus-buffer, 100 μl), MgSO_4_*aq.* (25 mM, 40 μl), PEG20,000*aq.* (12.5 wt%, 80 μl), TE buffer solutions of the dNTP mixture (2 mM per each nucleotide, 80 μl), primer 1 (10 μM, 20 mer, 30 μl), primer 2 (10 μM, 20 mer, 30 μl) and 1,164-bp DNA template (10 nM, 10 μl). The conveyer GV contained the following solution: deionized water (740 μl), PCR buffer (100 μl), MgSO_4_*aq.* (25 mM, 40 μl), PEG20,000*aq*. (12.5 wt%, 80 μl), SYBR Green I (1/10,000, 20 μl) and DNA polymerase (0.6 μM, 1.0 units per microlitre KOD-Plus-, 20 μl). As described above, the conveyer GV was fused with the target GV. The mixed GV dispersion was subjected to thermal cycling. The GVs that emitted fluorescence from the complex between SYBR Green I and amplified dsDNA were observed using differential interference contrast and fluorescence microscopies.

## Additional information

**How to cite this article:** Kurihara, K. *et al*. A recursive vesicle-based model protocell with a primitive model cell cycle. *Nat. Commun.* 6:8352 doi: 10.1038/ncomms9352 (2015).

## Supplementary Material

Supplementary Information Supplementary Figures 1-7
