# Supplementary material for: A recursive vesicle-based model protocell with a primitive model cell cycle
Source: Nat Commun. 2015 Sep 29;6:8352. doi: 10.1038/ncomms9352 (PMC4598553; doi:10.1038/ncomms9352)
Supplement: Supplementary Information — Supplementary Figures 1-7 [file ncomms9352-s1.pdf]

Supplementary Figures

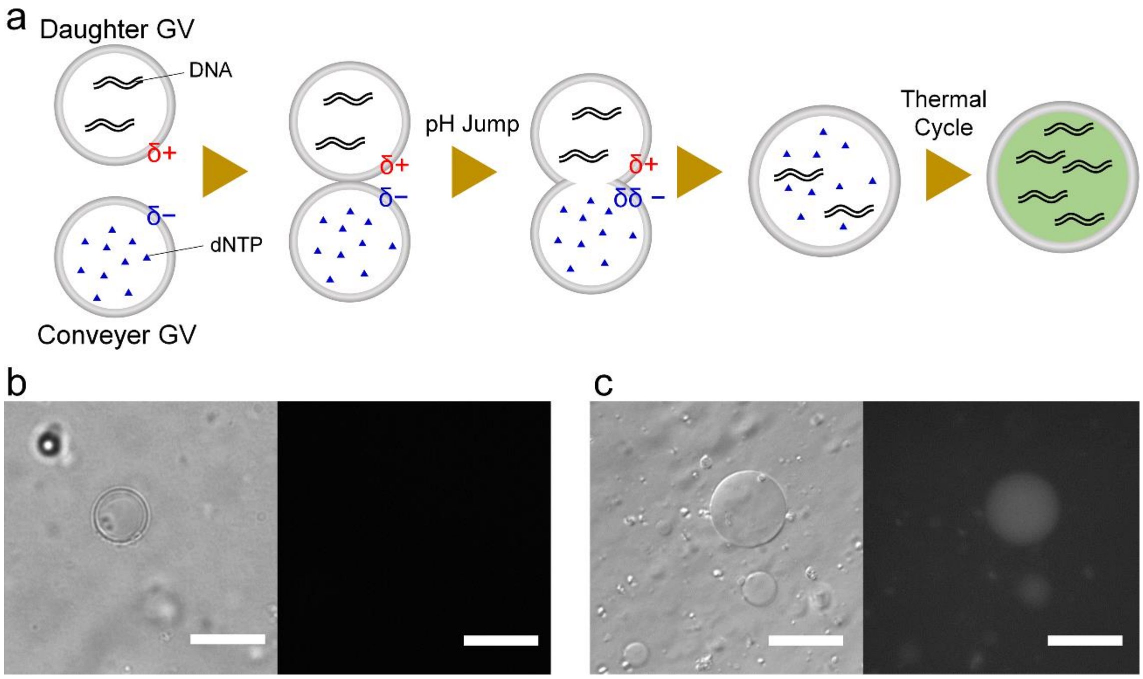

Supplementary Figure 1 | Model study of pH-induced vesicular delivery system. (a) Schematic illustration of DNA-amplification using transported dNTP. In this case, the surface charge of the daughter GV is positive, but pH-jump is still necessary for complete transportation of depleted substrates. (**b**) Images of differential interference contrast microscope (left) and fluorescence microscope (right) of fused GV before thermal cycles. (**c**) Fluorescence emission (right) after thermal cycles indicates amplification of dsDNA using transported dNTP. The scale bars indicate 50 μm.

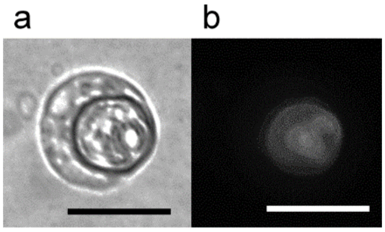

Supplementary Figure 2 | Adhesion between target GV and conveyer GV. (**a**) Images of differential interference contrast microscope after adhesion at pH = 3. (**b**) Images of fluorescence microscope after adhesion at pH = 3. The scale bars indicate 20 μm. When an acidic dispersion (pH = 3) of conveyer GVs with the membrane composition of POPC : POPG : C : Cholesterol : Rhod-DOPE = 25: 60: 10: 5: 0.1 (mol%), was added to a dispersion of target GVs containing membrane molecule **V** (the composition of the target GV was POPC: POPG: **V**: **C**: cholesterol = 30: 7.5: 55: 5: 2.5 mol), adhered GVs appeared 24 h after the mixing as observed by a phase contrast microscope. Fluorescence microscope image of the mixture indicated that only the membranes of adhered GVs emitted intense fluorescence light.

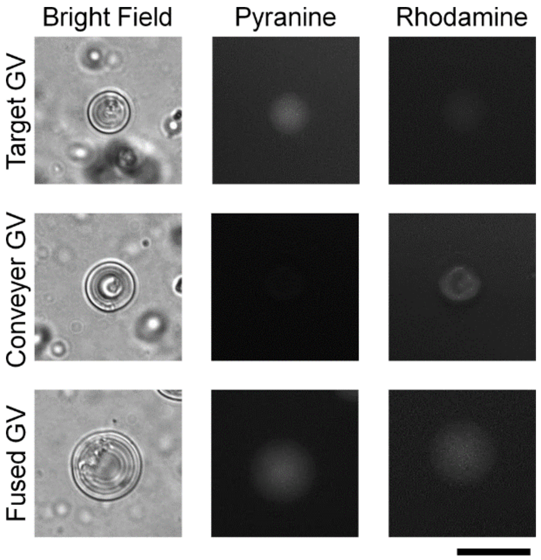

Supplementary Figure 3 | Proton impermeability of membranes of target GV. Differential interference microscope images of the target GV, conveyer GV and fused GV at pH = 3. The scale bar indicates 20 μm. The impermeability of the vesicular membrane against hydrochloric acid dissolved in an exterior water phase was examined using a pH sensitive polyanionic fluorophore, *i.e.* pyranine (8-hydroxy-1,3,6-pyrenetrisulfonate). Pyranine emits fluorescence at pH ~ 8 (λ_{ex} = 460 nm, λ_{em} = 510 nm) but no fluorescence at pH = 3. The target GV (1 mM, 1 mL) with the membrane composition of POPC : POPG : V : C : Cholesterol = 30 : 7.5 : 55 : 5 : 2.5 (mol%) was prepared by the freeze-dry method. The rehydration buffer solution contained pyranine (0.5 mM, pH ~ 8). The conveyer GV (1 mM, 1 mL) with the membrane composition of POPC : POPG : C : Cholesterol : Rhod-DOPE = 25 : 60 : 10 : 5 : 0.1 (mol%) was prepared, and the membrane was stained with Rhod-DOPE (λ_{ex} = 550 nm, λ_{em} = 570 nm). After mixing of the dispersions of target GV and conveyer GV, respectively, pH of the mixed dispersion was adjusted to 3 by dropping hydrochloric acid and was incubated for 24 hours. The fluorescence of GV was detected through a U-MWIB2 filter set for pyranine and through a MWIG2 filter set for Rhodamine. The target GV which contains pyranine in the inner water showed the green fluorescence at pH = 3 in being observed with the U-MWIB2 filter unit. The conveyer GV the membrane of which was stained by Rhodamine showed red fluorescence in being observed with the MWIG2 filter unit. The fused GV showed the both red and green fluorescence at pH = 3, indicating that the pH of inside the target GV maintain to be ~ 8.

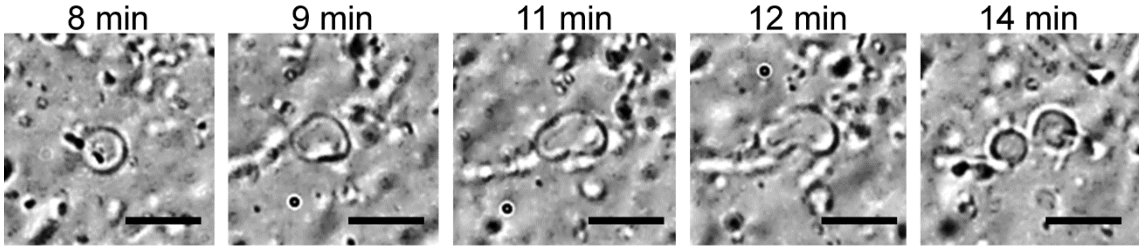

Supplementary Figure 4 | Division of DNA-amplified daughter GV after addition of V*. Sequential differential interference contrast microscope images of divisions of the daughter GV formed by the model experiment shown in Supplementary Fig.1. The scale bars indicate 20 μm.

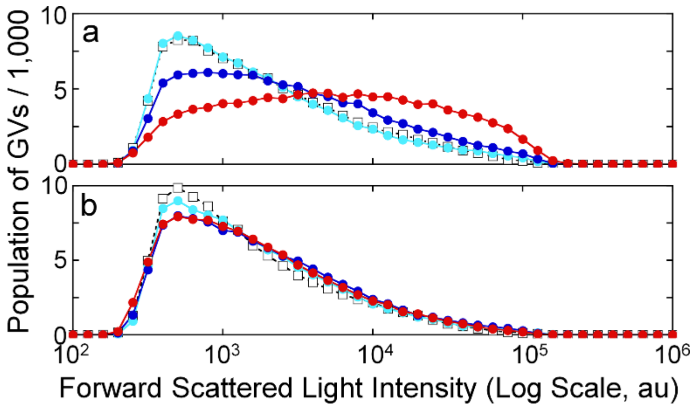

Supplementary Figure 5 | Statistical analysis on influence of fusion of target GV (dNTP-depleted GV) with conveyer GV filled with dNTP by Flow Cytometry. (**a**) Temporal changes of histograms of FSC (forward scattered light intensity) from GVs after addition of V* [0 min (turquoise), 60 m (blue), and 120 m (red)]. The histogram shows a distinct increase in the population (number of GVs) with FCS larger than10^4 (The forward scattered light intensity of 1×10^5 corresponds to a diameter of GV of 10 μm on the basis of the filtration experiment). **(b)** Histogram (**b**) is a negative control to **(a)**. In the negative experiment, target GV fused with empty conveyer GVs.

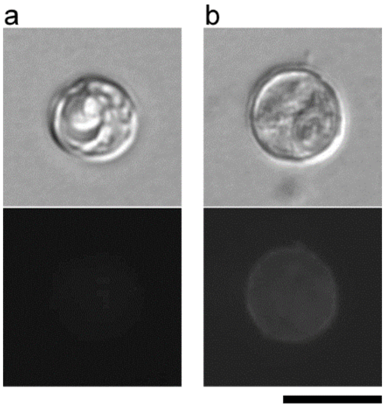

Supplementary Figure 6 | DNA-amplified GV using the transported DNA-polymerase after pH-induced fusion with conveyer GV. Differential interference contrast and fluorescence

microscope images of by the fused GV before (**a**) and after PCR (**b**). Scale bar indicates 20 μm.

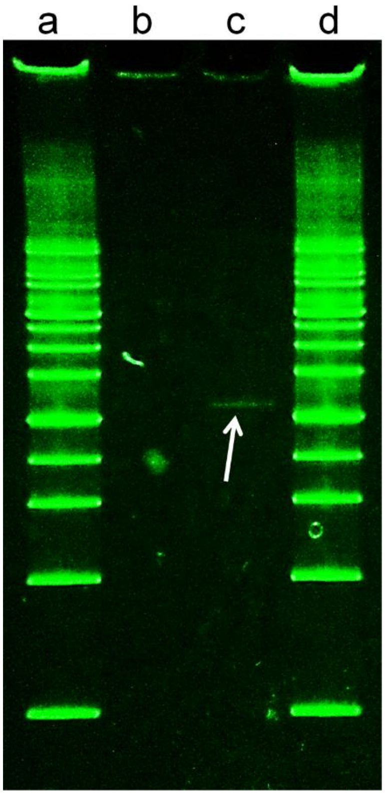

n

Supplementary Figure 7 | PAGE images of amplified DNA in fused GV after PCR. (**a**, **d**) 200 bp ladder marker (**b**) mixed GV at pH = 8.1 (**c**) fused GV at pH =3. White arrow indicates 1,164 bp amplified DNA
